# Prevalence of Transmitted Drug Resistance and Impact of Transmitted Resistance on Treatment Success in the German HIV-1 Seroconverter Cohort

**DOI:** 10.1371/journal.pone.0012718

**Published:** 2010-10-07

**Authors:** Barbara Bartmeyer, Claudia Kuecherer, Claudia Houareau, Johanna Werning, Kathrin Keeren, Sybille Somogyi, Christian Kollan, Heiko Jessen, Stephan Dupke, Osamah Hamouda, on behalf of the German HIV-1 Seroconverter Study Group

**Affiliations:** 1 HIV/AIDS, STD Unit, Department Infectious Disease Epidemiology, Robert Koch-Institute, Berlin, Germany; 2 Project HIV Variability and Molecular Epidemiology, Robert Koch-Institute, Berlin, Germany; 3 Gemeinschaftspraxis Jessen-Jessen-Stein, Berlin, Germany; 4 Gemeinschaftspraxis Dupke, Baumgarten, Carganico, Berlin, Germany; Erasmus Medical Center, Netherlands

## Abstract

**Background:**

The aim of this study is to analyse the prevalence of transmitted drug resistance, TDR, and the impact of TDR on treatment success in the German HIV-1 Seroconverter Cohort.

**Methods:**

Genotypic resistance analysis was performed in treatment-naïve study patients whose sample was available 1,312/1,564 (83.9% October 2008). A genotypic resistance result was obtained for 1,276/1,312 (97.3%). The resistance associated mutations were identified according to the surveillance drug resistance mutations list recommended for drug-naïve patients. Treatment success was determined as viral suppression below 500 copies/ml.

**Results:**

Prevalence of TDR was stable at a high level between 1996 and 2007 in the German HIV-1 Seroconverter Cohort (N = 158/1,276; 12.4%; CI_wilson_ 10.7–14.3; p _for trend_ = 0.25). NRTI resistance was predominant (7.5%) but decreased significantly over time (CI_Wilson_: 6.2–9.1, p_ for trend_ = 0.02). NNRTI resistance tended to increase over time (NNRTI: 3.5%; CI_Wilson_: 2.6–4.6; p_ for trend_  = 0.07), whereas PI resistance remained stable (PI: 3.0%; CI_Wilson_: 2.1–4.0; p_ for trend_  = 0.24). Resistance to all drug classes was frequently caused by singleton resistance mutations (NRTI 55.6%, PI 68.4%, NNRTI 99.1%). The majority of NRTI-resistant strains (79.8%) carried resistance-associated mutations selected by the thymidine analogues zidovudine and stavudine. Preferably 2NRTI/1PIr combinations were prescribed as first line regimen in patients with resistant HIV as well as in patients with susceptible strains (susceptible 45.3%; 173/382 vs. resistant 65.5%; 40/61). The majority of patients in both groups were treated successfully within the first year after ART-initiation (susceptible: 89.9%; 62/69; resistant: 7/9; 77.8%).

**Conclusion:**

Overall prevalence of TDR remained stable at a high level but trends of resistance against drug classes differed over time. The significant decrease of NRTI-resistance in patients newly infected with HIV might be related to the introduction of novel antiretroviral drugs and a wider use of genotypic resistance analysis prior to treatment initiation.

## Introduction

Testing for HIV resistance to antiretroviral drugs in plasma is a rational supplement to guide clinicians in their therapeutic choice for patients receiving antiretroviral therapy, ART. Problems of drug failure related to drug resistance as a consequence of individual pharmacogenetics and adherence problems rendering drug levels suboptimal remains a major obstacle for clinicians to choose an effective antiretroviral regime. Once resistant strains of HIV are selected in the host population, they can be transmitted to new hosts and spread in the population at risk. Resistance-associated mutations of HIV have been documented for each of the different classes of antiretroviral drugs [1], [2], [3]. After a continuous increase of transmitted drug resistance (TDR) over several years in Western Europe stable or decreasing trends of TDR were reported [4], [5], [6], [7], [8].

Studies of the impact of primary resistance on treatment response [9], [10] are limited by the fact that many have been undertaken before routine baseline resistance testing became common, and therefore inactive drugs were often utilised, and associated with either reduced treatment response, or no effect. Data on the impact of genotypic resistance testing on the composition of first-line therapy are scarce [11], [12], [13]. Some studies indicate variation among treatment strategies of physicians. Differences in HIV treatment practices have been attributed not only to medical factors but also to non-medical factors such as patient adherence [14], [15], [16]. The aim of this study was to investigate the prevalence of TDR and the impact of the infection with resistant HIV on treatment success in patients with a known or well estimated date of seroconversion as best approximation for the date of infection in the German HIV-1 Seroconverter cohort.

## Methods

### Study population

The study was approved by the local ethical committee of the Charité, University Medicine, Berlin, Germany. Written informed consent was given by all patients. The HIV-1 Seroconverter Study is a nationwide multicentre observational study based on an open cohort of HIV-1-infected persons for whom the date of seroconversion is known (1. acute seroconverters) or can be estimated (2. documented seroconverters).

1. Acute seroconverters: laboratory diagnostic criteria for an acute seroconversion were i) detectable HIV-1 RNA (or p24 antigen) combined with a negative or indeterminate ELISA result or ii) reactive HIV-1-ELISA combined with a negative or indeterminate immunoblot-result followed by confirmation of complete seroconversion within 6 months. In persons recruited during seroconversion the date of the first reactive test was used as an approximation for the infection date.
2. Documented seroconverters: individuals with a last negative and a first positive HIV-antibody test with a maximum three year interval were recruited for the study. In documented seroconverters the mid-point between the dates of the last negative and the first positive HIV-antibody test was used to estimate the time of infection.

As we performed a long term observational study, various commercial kits to determine viral load (VL) with different detection limits were used over time. In this analysis viral suppression was determined as treatment success in case of viraemic suppression to at least 500 copies/ml including any category of low-level viral load measurement (<400, <50 and <40 copies/ml). Two consecutive viral load measurements within 5 to 12 months after initiation of first line therapy were mandatory for inclusion into the analysis of treatment success. Two or more consecutive viral load measurements <500 copies/ml were categorised as virological failure. We defined 3 months as a minimum duration of first line treatment to analyse for treatment success.

### Genotypic resistance testing

At enrolment baseline blood samples were collected from each individual. Genotypic resistance testing was only performed for antiretroviral-naïve patients. The ViroSeq™ HIV-1 Genotyping System was applied according to the instructions of the manufacturer (Abbott, Germany). Alternatively, viral RNA was extracted from plasma using the viral RNA Kit (Qiagen, Germany) and reverse transcribed (70 µl plasma equivalents) with Superscript II (Invitrogen, Germany) and amplified with the Expand high fidelity PCR system (Boehringer, Germany). The 1.5-kb *pol* fragments encoding the complete protease (99 amino acids) and reverse transcriptase (1–296 amino acids) were directly sequenced using in-house primers. This method has already been described elsewhere [12], [17]. For the purpose of this study, the surveillance drug resistance mutations list (Bennett D, Camacho R, Otela D, Kuritzkes D, Fleury H et al., 2009) was used for identification of resistance associated-mutations [18], [19] and the prevalence of TDR. TDR was considered if any of these mutations was present and the prevalence of TDR was calculated by year of seroconversion.

### Statistical analyses

Proportions were calculated with a 95% Wilson score confidence interval based on a binomial distribution. Median values were given with the interquartile ranges (IQR). –Due to low numbers Fishers's exact test was used to compare proportions. The Mann-Whitney-U-test (MWT) was used to compare age, viral loads and CD4-cell-counts. Trends in the prevalence of TDR were calculated by logistic regression equivalent to the Cox-Armitage trend test. Kaplan-Meier analysis was used for the comparison of duration of first line regimen. Two consecutive viral load measurements within 5 to 12 months after initiation of first line therapy were mandatory for inclusion into the analysis of treatment success. Two or more consecutive viral load measurements above 500 copies/ml were categorised as virological failure. Therapeutic success was defined as viral suppression below the detection limit of at least ≤500 copies/ml. We defined 3 months as a minimum duration of first line treatment. All p-values are two sided, and a p-value of <0.05 was considered significant. All data were analysed using SPSS 17.0.

## Results

### Population characteristics

Of 1,564 HIV-positive patients with a known or estimated date of seroconversion 1,312 primary samples from drug-naïve patients were available and included into the analysis. All study participants seroconverted between 01.01.1996 and 31.12.2007. Genotyping was performed in 1,276/1,312 available samples of treatment naive patients (97.3%). There was no bias between the seroconverters genotyped and the group of patients not included (no sample available or already treated at study entry with respect to exposure categories etc (data not shown). Nearly two thirds of patients were documented seroconverters (845/1,276; 66.2%; table 1). 33.8% were acute seroconverters (431/1,267) defined by laboratory criteria. 95.0% of the patients were male (1,212/1,267), 64 patients were female. In Germany data about route of transmission in HIV cases reported through the routine national surveillance system were documented in 85% of all patients newly diagnosed with HIV-1 infection (date of infection not known).

**Table 1:** Characteristics of patients.

	Total	resistant HIV^a^	susceptible HIV	OR (95%CI#)	p-Value
Drug-naive patients, first sample available, no. (%)	1276	158 (12.4)	1118 (87.6)		
Median age at sc& [years] (IQR§)	33 (28.0–38.0)	33.5 (27.8–38.3)	33.0 (28.0–38.0)	-	0.7^p^
Sex, no. (%)					
men	1212 (95.0)	151 (95.6)	1061 (94.9)	1.15 (0.52–2.59)	0.85 n
women	64 (5.0)	7 (4.4)	57 (5.1)		
Exposure category, no. (%)MSM	1119 (87.7)	140 (88.6)	979 (87.6)	1.1 (0.66–1.86)	0.80^n^
Heterosexual contacts	78 (6.1)	8 (5.1)	70 (6.3)	0.80 (0.38–1.69)	0.72 n
High prevalence countries	26 (2.0)	1 (0.6)	25 (2.2)	0.28 (0.04–2.07)	0.24 n
IVD	21 (1.7)	1 (0.6)	20 (1.8)	0.35 (0.05–2.62)	0.50 n
unknown	32 (2.5)	8 (5.1)	24 (2.1)	2.43 (1.07–5.51)	0.05* n
*pol-*subtype B, no. (%)	1182 (92.6)	154 (97.5)	1028 (91.9)	3.06 (1.16–8.08)	0.009** n
*pol-*subtype non-B, no. (%)	94 (7.4)	4 (2.5)	90 (8.1)		
Median viral load at sc [log_10_/ml] (IQR)	5.17 (4.4–5.9)	5.05 (4.4–5.6)	5.21 (4.4–5.9)	-	0.56 p
Median CD4^+^ cell count at sc [n/µl] (IQR)	518.5 (384.3–668.5)	555.0 (407.5–734.0)	515 (379.0–663.0)	-	0.35 p
Median duration of first-line regimen[days] (IQR)	180.0 (49.0–411.0)	144.0 (48.0–365.5)	182.0 (49.0–431.8)	-	0.56 p
Documented seroconversion, no. (%)	845 (66.2)	103 (65.2)	742 (66.7)		
Median viral load at sc [log_10_/ml] (IQR)	4.81 (3.9–5.5)	4.71 (4.4–5.0)	4.86 (3.9–5.5)	-	0.89 p
Median CD4^+^ cell count at sc [n/µl] (IQR)	545.0 (401.0–732.5)	537.5 (390.3–778.5)	545.0 (395.0–732.0)	-	0.97 p
Acute seroconversion, no. (%)	431 (33.8)	55 (34.8)	376 (33.6)		
Median viral load at sc [log_10_/ml] (IQR)	5.32 (4.6–5.9)	5.14 (4.4–5.7)	5.35 (4.6–5.9)	-	0.26 p
Median CD4^+^ cell count at sc [n/µl] (IQR)	509.0 (373.0–651.0)	555.0 (407.0–726.0)	504.5 (364.0–646.0)	-	0.22 p

a TDR according to Bennett et al., 2009,

& sc: seroconversion,

# CI: 95% confidence intervals,

§ IQR: interquartile ranges;

*statistically significant results (p<0.05);

**highly statistically significant results (p<0.01);

p Mann-Whitney - U Test;

n Fisher exact test.

65% were MSM, 17% were heterosexuals, 12% of the individuals originated from high prevalence countries. Intravenous drug use was reported for 5% of all reported HIV cases in 2008 (Epidemiological Bulletin, May 2009, Robert Koch-Institute, Berlin, Germany). Concordantly with all reported HIV-1 cases in Germany, the predominant route of transmission in the German HIV-1 Seroconverter cohort was sex between men, MSM (85.2%). Hence, our study has a bias towards MSM comparing to the national HIV surveillance data. However, the proportion of the transmission route is comparable to the national surveillance data.

NonB subtype was observed in 7.4% of the study participants. CD4 cell counts/µl and plasma viral load measured at time of seroconversion were 510 cells/µl (median; IQR: 380–652) and 5.17 log_10_/ml (median; IQR: 4.41–5.88), respectively (table 1). Antiretroviral therapy was initiated in 34.7% (443/1,267) of seroconverters. The median duration of the first-line regimen was 180 days (IQR 49–411).

Most patients who used their first-line regimen less than 49 days changed substances within one day (57.8%; 63/109). Changes within drug classes appeared in 39.7% of these patients (25/63). Unfortunately, reasons for regimen switches were not systematically documented by the practitioners. However, 24 of these 63 patients with a prescribed 2NRTI/1NNRTI regimen and 23 of 63 patients with a 2NRTI/1PIr regimen changed to another drug class. 30.3% (n = 33) of the 109 patients dropped out and no further information was available. The first-line regimen was censored by the date of data extraction for this manuscript in 11 cases (10.1%). For the remaining 1.8% (n = 2) no further treatment information was available.

### Genotypic resistance analysis

#### Prevalence and epidemiological trends in transmission of drug resistance

**Figure 1:**
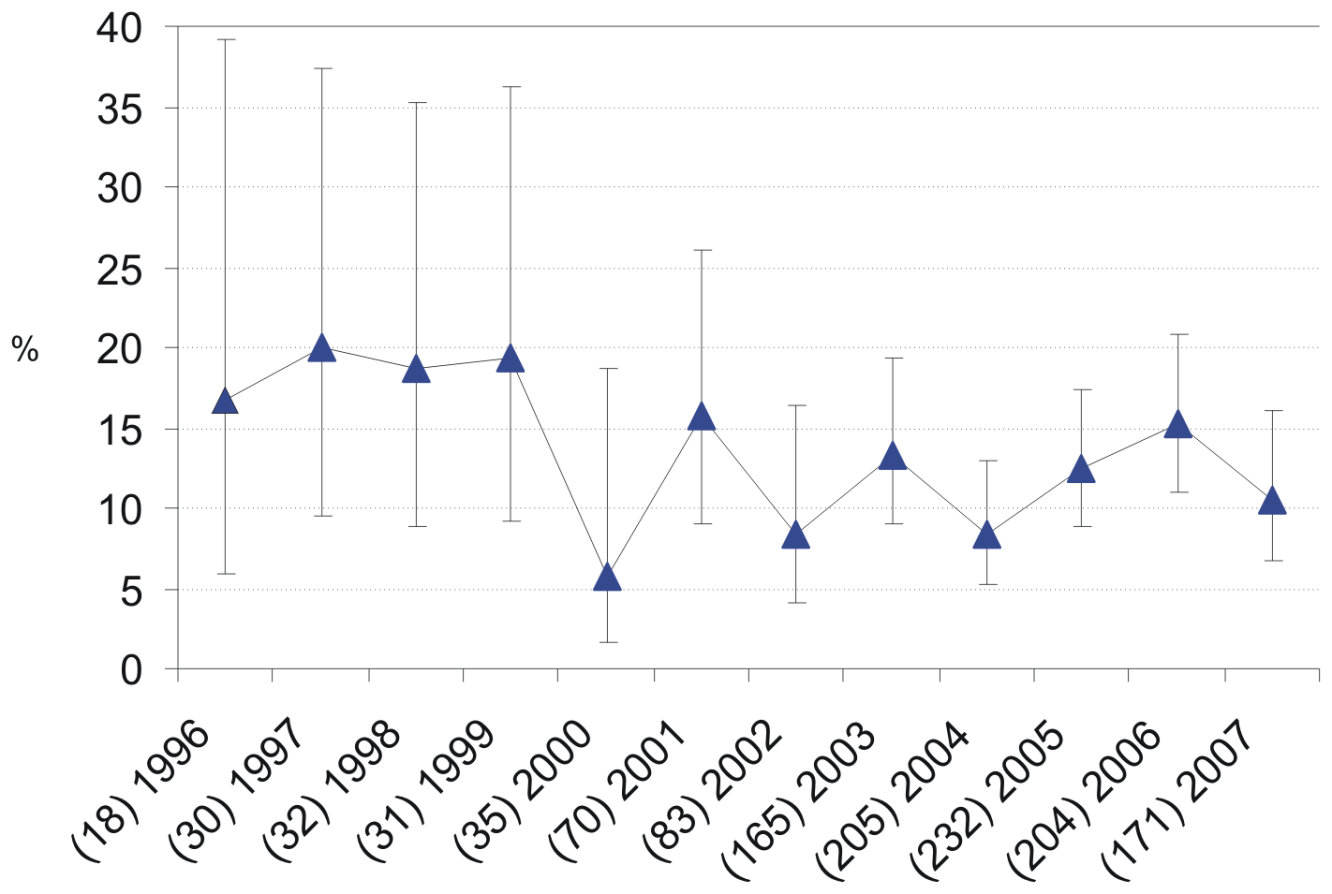
Overall prevalence of TDR in the German HIV-1 Seroconverter Cohort 1997–2007. The prevalence of TDR (%) is plotted by year of seroconversion (Bennett et al. 2009; 12.4%; CI 10.4–14.3; p *_for trend_* = 0.25. Numbers of genotyped HIV-infections were indicated in brackets before the year of seroconversion.

**Figure 2:**
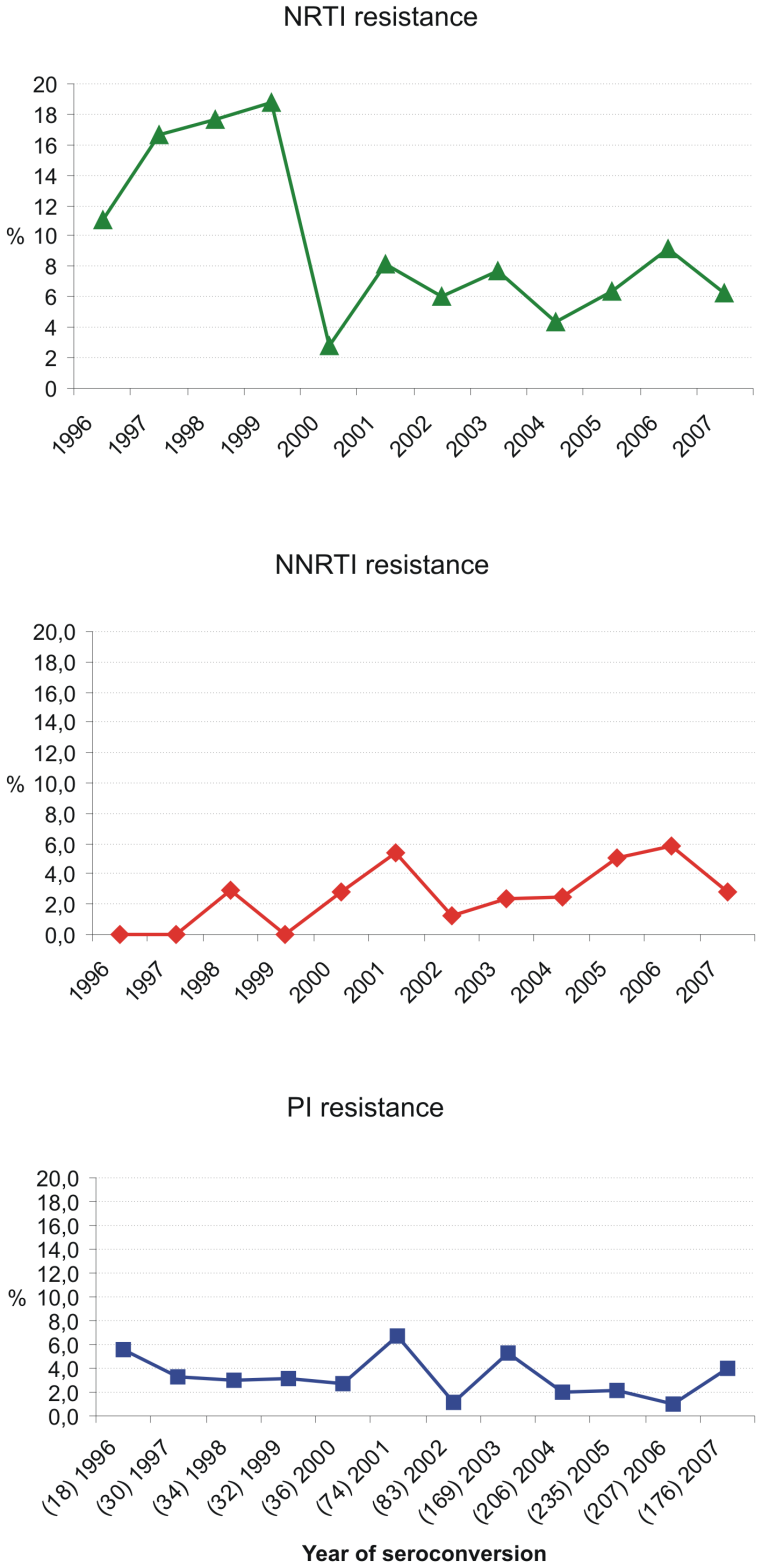
Proportion of TDR in the drug classes NRTI, NNRTI and PI by year of seroconversion. Resistance to individual drug classes was summed up for strains with single, dual- and multi-drug resistance to calculate the prevalence of HIV resistance to the three different drug classes. NRTI resistance decreased significantly over time (p *_for trend_*  = 0.02). NNRTI resistance increased over time (p *_for trend_*  = 0.07). PI resistance remained stable over time (p *_for trend_*  = 0.24). The cumulated numbers which where used to calculate the prevalence of each of the drug classes is indicated in brackets together with the year of seroconversion in the lowest panel of the figure (PI).

Primary mutations according to the SDRM list of Bennett et al. [18], [20], [21] were identified in 158/1,276 (12.4%) of viral strains. Overall, the prevalence of transmitted drug resistance remained rather stable at 12.4% (CI_Wilson_: 10.7–14.3) during the period of observation (*p_ for trend over time 1996–2007_* = 0.25) (figure 1). NRTI associated resistance (single class resistance) was identified most frequently (6.3%; 80/1276; CI_Wilson_: 5.0–7.8). Followed by 2.4% NNRTI resistance (30/1276; CI_Wilson_: 1.6–3.4) and 2.1% PI resistance (27/1276; CI_Wilson_: 1.4–3.1). Dual- and multi-class resistance was only seen in 1.4% and 0.2% of patients with TDR, respectively. NRTI resistance reached a mean prevalence of 7.5% (CI_Wilson_: 6.2–9.1), if the numbers were cumulated from all categories of transmitted resistance classes, e.g. mono-, dual- and multi-resistant HIV (NNRTI resistance 3.5% CI_Wilson_: 2.6–4.6; PI resistance 2.9% CI_Wilson_: 2.1–4.0). Prevalence of transmitted NRTI resistance dropped between 1999 and 2000 resulting in an overall declining trend (NRTI: *p_ for trend over time 1996–2007_*  = 0.02) (fig. 2). In contrast, prevalence of NNRTI resistance tended to increase over time (*p_ for trend_* = 0.07). Overall PI resistance remained stable among the study population genotyped over the time (*p_ for trend_* = 0.24; figure 2).

#### Factors associated with transmitted drug resistance

The prevalence of TDR was highest among patients exposed to HIV-1 through sex between men (12.5%, CI: 10.7–14.9; heterosexuals 10.3%; CI: 5.3–19.0; HPL 3.9%; CI: 0.7–18.9; and IVDU 4.8%; CI: 0.9–22.7). Men acquired TDR more frequently than women (12.5%, CI: 10.7–14.4; vs. 10.9%, CI: 5.4–20.9), respectively. The median age at seroconversion did not differ between patients with or without TDR (33.5; IQR 27.8–38.3 *vs*. 33.0; IQR 28.0–38.0; p_MWT_  = 0.7) (table 1). In a univariate analysis, there was no evidence to suggest, that TDR was associated with sex (OR  = 1.15; CI: 0.5–2.6; p_Fisher Exact_ = 0.9). Being infected with subtype B virus was associated with a significantly higher risk to acquire resistant HIV strains (OR = 3.06; CI: 1.16–8.08; p_Fisher Exact_  = 0.009) and the exposure category unknown is significantly more frequently reported in patients with resistant HIV strains (OR = 2.43; CI: 1.07–5.51; p_Fisher Exact_  = 0.05; table 1).

#### Transmitted drug resistance is frequently caused by single resistance mutations

TDR was mostly related to viruses carrying single resistance mutations (figure S4). The majority of NNRTI resistance, 99.1% was caused by a singleton resistance mutation, followed by 68.4% singleton PI resistance mutations and 55.6% singleton NRTI resistance mutations. TAM mediated NRTI resistance was due in 54.4% to more than one TAM present in the same viral genome.

#### Dominance of NRTI resistance mutations, TAMs and T215 revertant substitutions

**Figure 3:**
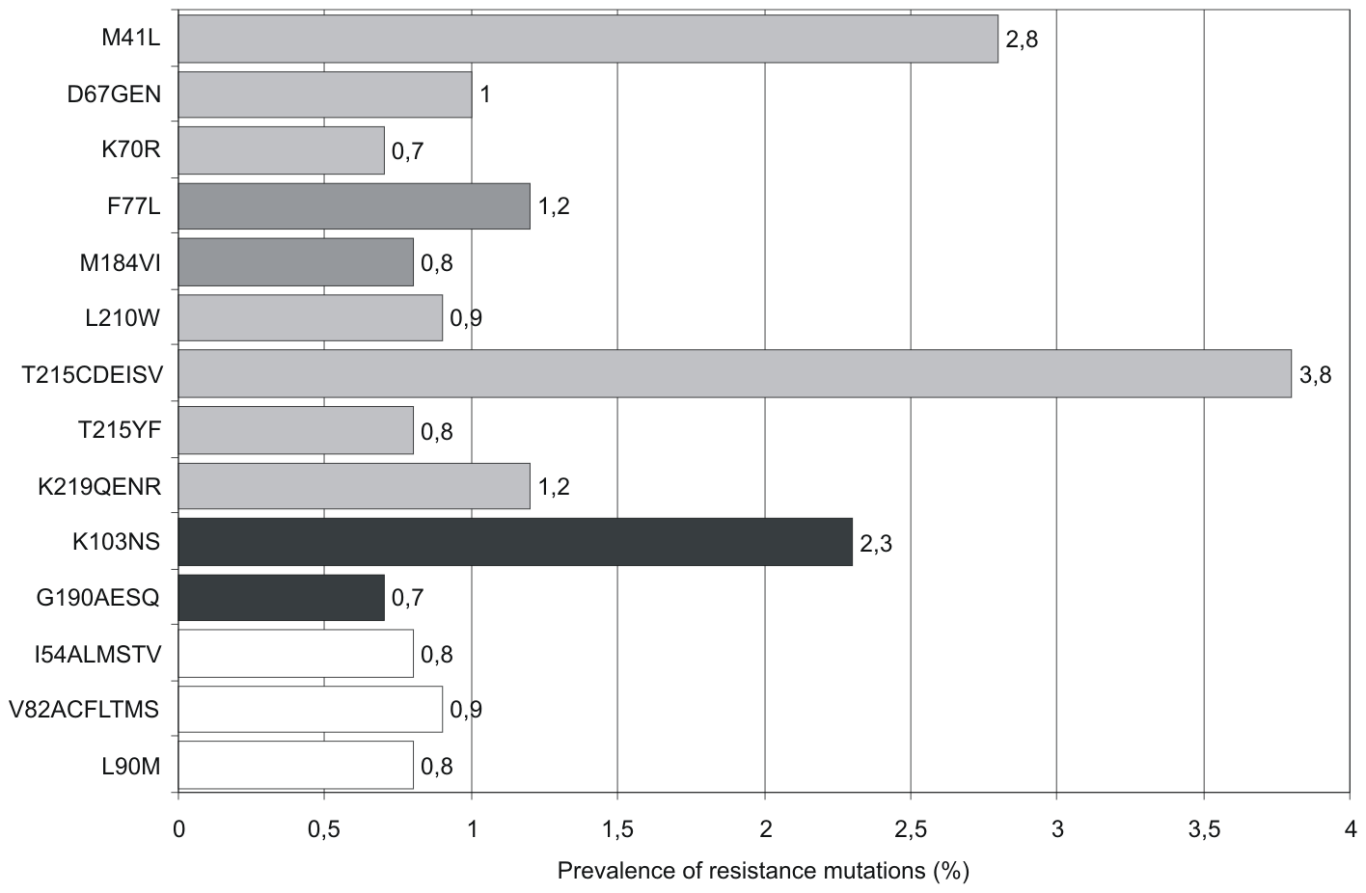
Prevalence of resistance mutations identified between 1997 and 2007. HIV resistance mutations (SDRM list) which occurred at a prevalence of ≥0.7% in treatment naïve seroconverters were depicted. The RT mutations K65R, T69i, L74VI, V75AMTS, F116Y, Q151M, K219R; L100I, K101EP, P225H and the PR mutation L23I; I50VL, L76V never occurred. Bars in light grey: TAMS; Bars in dark grey: NRTI resistance mutations, black bars: NNRTI resistance mutations, white bars: PI resistance mutations.

The majority of the NRTI resistant strains (79.8%) carried thymidine analogue resistance mutations (TAMs) selected by zidovudine and stavudine. Among the TAMs, revertant amino acid substitutions at position 215 of the reverse transcriptase were most prevalent (74.7%). Despite the significant decrease of transmitted NRTI resistance there is no clear cut decrease of either the prevalence of TAMs or the T215 revertant substitions (figure S1). T215 revertant substitutions were by far the most prevalent resistance mutations (3.8%) in the study population (figure 3).

### Composition of antiretroviral first-line treatment in patients with susceptible and resistant HIV

Analysis of treatment and treatment response was performed on the basis of resistance data according to the SDRM list of mutations. First-line therapy was initiated in 382/1,118 (34.2%) patients with susceptible strains and in 61/158 (38.6%) patients with TDR associated mutations.

#### First-line therapy of patients with susceptible genotype

First-line therapy was initiated in 382 patients with susceptible HIV strains and lasted for 182.0 days (median, IQR: 49.0–431.8). A Kaplan-Meier analysis revealed no significant difference in duration of first-line therapy comparing susceptible and resistant strains (χ^2^_Log Rank_  = 0.004; p = 0.95). Time between seroconversion and ART initiation was 327 days (median, IQR, 66.8–652.5) and Kaplan-Meier showed no significant difference in comparison to patients with resistant HIV strains (χ^2^_Log Rank_  = 0.454; p = 0.50). CD4 cell count measurements reported at the start of therapy were 335 cells/µl (median, IQR: 215.0–511.5) and plasma HIV-RNA measurement of 5.3 log_10_/ml (median, IQR: 4.7–5.8). Any PI containing first line therapy was prescribed in 60.5% (231/382) of the patients with susceptible genotype. Predominantly, a 2 NRTI/1 PIr regimen was used as first-line therapy in 45.3% (173/382) of the patients. 34.3% (131/382) of individuals with susceptible strains received a NRTI/NNRTI first-line therapy. Any NNRTI comprising first-line therapy was initiated in 38.7% (148/382) of the patients. Efavirenz was preferred to nevirapine (92/148; 62.2% *vs*. 55/148; 37.2%, respectively).

#### First line therapy of patients with resistant genotype

First line therapy was initiated in 61 patients (61/158) with TDR. Time between seroconversion and start of first-line therapy was 448 days (median, IQR 162–660). The durability of the first-line regimen in this group was 144 days (median, IQR: 48–365). CD4 cell counts measured at time of antiretroviral treatment initiation were 312 cells/µl median and did not differ significantly to the susceptible group (IQR: 200–458; p_MWT_ = 0.48). Also, plasma viral load was not significantly lower in patients with resistant strains compared to initial viral load measurements in the susceptible group (median, log_10_/ml: 5.2; IQR: 4.6–5.5; p_MWT_ = 0.36). Comparing VL between acute seroconverters with and without TDR, no significant difference was observed. First-line regimen containing any PI were used in 37/61 (60.7%) patients with resistant HIV. Any NNRTI was used in 24/61 (39.3%) patients in the TDR group. Efavirenz was again the preferred NNRTI compared to nevirapine in patients with TDR (16/24; 66.7% *vs.* 6/24; 25.0%).

### Treatment success in patients with susceptible and resistant HIV

#### Viral suppression in patients with susceptible phenotype

Data about any viral load measurement during first year of first-line therapy were available for 271/382 (71%) of the patients with susceptible genotype. After applying the minimum duration of first-line therapy, e.g. 3 months, the patients included were 184/252 (73%). Consecutive viral load measurements within 5 to 12 months after treatment initiation were scarce. Data eligible for the analysis of treatment success were available only from 37.5% (69/184) of antiretroviral treated patients in the cohort. The majority of these patients with available viral load data (89.9%, 62/69) were treated successfully. Only 2.9% (2/69) of individuals did not reach the viral load limit (≤500 copies/ml) between 5–12 months after start of therapy. 5/69 (7.2%) patients were reported to have had two to three detectable viral load measurements after viraemic suppression (table 2).

**Table 2:** Treatment success of patients with susceptible and resistant HIV 5–12 months after ART- initiation.

Characteristics of patients on first line therapy (FLT)	Predicted phenotype	
	Susceptible	Resistant
Viral load (VL) data available within 12 months of FLT and >1 VL report	69/184 (37.5%)	9/40 (22.5%)
Failure: 2 or more VL >500 copies/ml	2/69 (2.9%)	2/9 (22.2%)
Success: VL constant ≤500 copies/ml	62/69 (89.9%)	7/9 (77.8%)
Any detectable viral load measurements: 1 VL >500 copies/ml	5/69 (7.2%)	none

#### Viral suppression in patients with resistant phenotype

Data about any viral load measurement data during first year of first-line therapy – taking the minimum duration of first line therapy into account - were available for 9/40 (22.5%) of patients with resistant HIV (table 2). Therapeutic success was achieved in the majority of treated patients (7/9, 77.8%). Two patients did not reach viraemic suppression (≤500 copies/ml) between 5–12 months after start of therapy.

#### Time trend in treatment response in patients with susceptible phenotype

Referring to the subpopulation entered into the analysis of treatment success, we performed an analysis on time trends in response to therapy solely in the group of patients infected with susceptible HIV strains (69 patients). We excluded the group of patients infected with resistant HIV due to small numbers (9 patients were entered into analysis on treatment success).

We used a logistic regression equivalent to the Cox-Armitage trend test. In this logistic regression analysis we applied as dependent variable binary coded treatment success and as covariant ‘Year of start of first line therapy’ (categories were: 1997–2000 = 0; 2001–2004 = 1; 2005–2007 = 2). The result indicates that with the higher the category the more likely to have treatment success (β = 0.32; p = 0.12; OR = 1.37; CI: 0.93–2.04). However note, that the result is not significant at the alpha = 0.05 level.

## Discussion

In contrast to trends of TDR reported in other European cohorts [4], [6], [22], [23], [24], [25], [26], prevalence of TDR was stable at a high level during a decade of observation (1997–2007) in the German HIV-1 Seroconverter cohort [18], [19]. Estimates of the prevalence of TDR may vary in cohorts because of differences in the study design, geographical location, definitions and classifications of TDR and composition of the study population. Prevalence of resistance is higher in patients with a known date of infection than in patients with an established but unknown date of HIV-infection. This was supported by lower prevalence of TDR in patients chronically infected with HIV as reported in a prospective multi centre cohort in the state of Nordrhine-Westfalia in Germany (9%; 95% CI 7.1–10.9) [27]. Reversion to wild type of resistance mutations affecting viral fitness from the dominant quasispecies over time is supposed to be the reason for the lower prevalence of TDR in chronically infected patients. As an example the mutations M184V and T215Y/F known to strongly reduce viral replication efficiency were never observed in chronically infected patients [30] in contrast to the population of newly infected HIV-1 seroconverters analysed in this study (M184V n = 10 and T215YF n = 10, espectively; figure 3).

The significant decrease of NRTI-related resistance over time (p = 0.02) in this population might have been caused by several factors. The reduction of the intensive use of mono- and dual therapies in previous years and the reduced replication capacity of viruses harbouring NRTI and multi drug resistance are associated with the reduced prevalence of NRTI resistance [28], [29]. Additionally, improved treatment strategies, low tolerance of detectable viral load, and prompt management of treatment failure by the physicians might have lead to a reduction of NRTI resistance in treatment experienced persons subsequently influencing the spread of TDR in patients recently infected with HIV. Indeed, the decrease of NRTI resistance coincided with a decrease of the prevalence of the lamivudine selected mutation M184V and the TAMs T215YF, K219Q, K70R, in patients newly infected with HIV (figure S2). In contrast, some distinct NNRTI mutations (P225H, G190AS and Y181C) and also HIV strains carrying more than one NNRTI mutation were only observed since 2003/2004 and might contribute to the increase in NNRTI-resistance (figure S3), whereas the prevalence of the prominent NNRTI mutation K103NS (2.3%) is stable during the observation period (figure S3).

The overall prevalence of viruses with genotypic resistance in recently HIV-infected individuals did not vary over time [30], although the distribution of resistance between individual drug classes has changed as also shown in our study. The most common NRTI-resistance mutations detected in treatment-naïve patients were M184V/I and TAMs, reflecting the extensive use of 3TC, ZDV and d4T and the use of mono and dual NRTI-therapy in the pre- and early HAART era. The T215 revertant was the most prevalent NRTI related mutation among patients with primary resistance [13], [31]. These strains are phenotypically susceptible to zidovudine and stavudine *in vitro* but are able to acquire T215Y more rapidly than the wild type under the selective pressure of zidovudine [32]–[33]. Evidence exists that T215 revertants replicate as efficiently as the wild-type virus or even gained improved fitness and growth advantage compared to T215Y/F [34], [35], [36].

Many treatment options are available for the initial antiretroviral regimen of patients infected with HIV [37], [38]. Reports describing the inferior treatment outcome in patients harbouring TDR, were frequently published [2], [22], [39]. If resistance testing is not possible or the patients needs to commence treatment before resistance results are available in case of low CD4 numbers, it may be advisable to initiate treatment with a boosted PI-regimen [40], Taking into account that boosted PIs have a higher barrier to develop resistance, an accumulation of mutations is required to induce PI-related resistance. As observed in this cohort, the majority of the patients either carrying susceptible or resistant HIV strains, was treated with any PI comprising regimen as the first-line regimen. Mostly, a ritonavir boosted lopinavir/NRTI first-line regimen was preferred, reflecting the extended therapeutic options since PIs of the second generation were available for treatment.

It was previously assumed, that TDR detected at HIV-diagnosis would not impact the course of infection as fitter wild-type strains would become the dominant quasispecies. It is now well known, that TDR can persist for years as dominant quasispecies [41] and for even longer in a minority of the viral quasispecies in plasma (RNA) and as archived resistance within proviral DNA copies in the genome of peripheral blood mononuclear cells (PBMCs) [34] and other target cells of HIV. Therefore treatment response might be influenced by TDR even after many years, resulting in ongoing viral replication under selective pressure and promoting the evolution of further resistant variants [42]. First results in this study showed that treatment response was successful in patients with TDR bearing in mind that only small numbers of viral load measurements after start of therapy were available [10].

More than half of the patients in both groups (resistant 77.8%; susceptible 89.9%) were reported to be under detection limit within 5–12 months (≤500 copies/ml) after start of antiretroviral treatment. A long-term observation of the changes in the composition of individual antiretroviral regimen and monitoring of the treatment outcome is necessary to assess treatment success adequately. As reported in other studies, the presence of revertants, which were identified at a high prevalence in this study, seems to have a negative impact on virological response [6], [35], [43]. However, the results of this study underline the fact that most persons with TDR have good treatment outcome by using resistance testing to guide the choice of a first-line regimen [13], [44].

Our findings reinforce that a broad and representative HIV resistance surveillance network between virologists, practitioners, clinicians and patients has to be installed to intensify the epidemiological knowledge about the transmission of resistant HIV, genotypic resistance testing frequencies, and treatment response in patients carrying resistant strain.

## Supporting Information

Figure S1 Prevalence of TAMs and NRTI resistance mutations by year of seroconversion. The prevalences of TAMs and NRTI resistance mutations (SDRM list) were calculated by year of seroconversion.
(0.23 MB TIF)

Figure S2 Prevalence of NRTI resistance mutations by year of seroconversion. The prevalences of each of TAMs and other NRTI resistance mutations (SDRM list) were calculated per year of seroconversion. A TAMs. Never observed: K219R. B NRTI resistance mutations other than TAMs. Never observed: K65R, T69i, L74V, V75AMTS, F116Y, Q151M.
(0.88 MB TIF)

Figure S3 Prevalence of NNRTI and PI resistance mutations by year of seroconversion. The prevalences of each of the NNRTI and PI resistance mutations (SDRM list) were calculated by year of seroconversion. A NNRTI resistance mutations. Never observed: L100I, K101E, V106M, Y181I, G190EQ, M230L (P236L not included). B PI resistance mutations. Occurred once: L24I, V32I, G48V, G73ACST.
(0.73 MB TIF)

Figure S4 Prevalence of TDR caused by one or more resistance mutations in the HIV genome by year of seroconversion.
(0.51 MB TIF)
