# Supplementary figures and images for: Prevalence of Transmitted Drug Resistance and Impact of Transmitted Resistance on Treatment Success in the German HIV-1 Seroconverter Cohort
Source: PLoS One. 2010 Oct 7;5(10):e12718. doi: 10.1371/journal.pone.0012718 (PMC2951346; doi:10.1371/journal.pone.0012718)

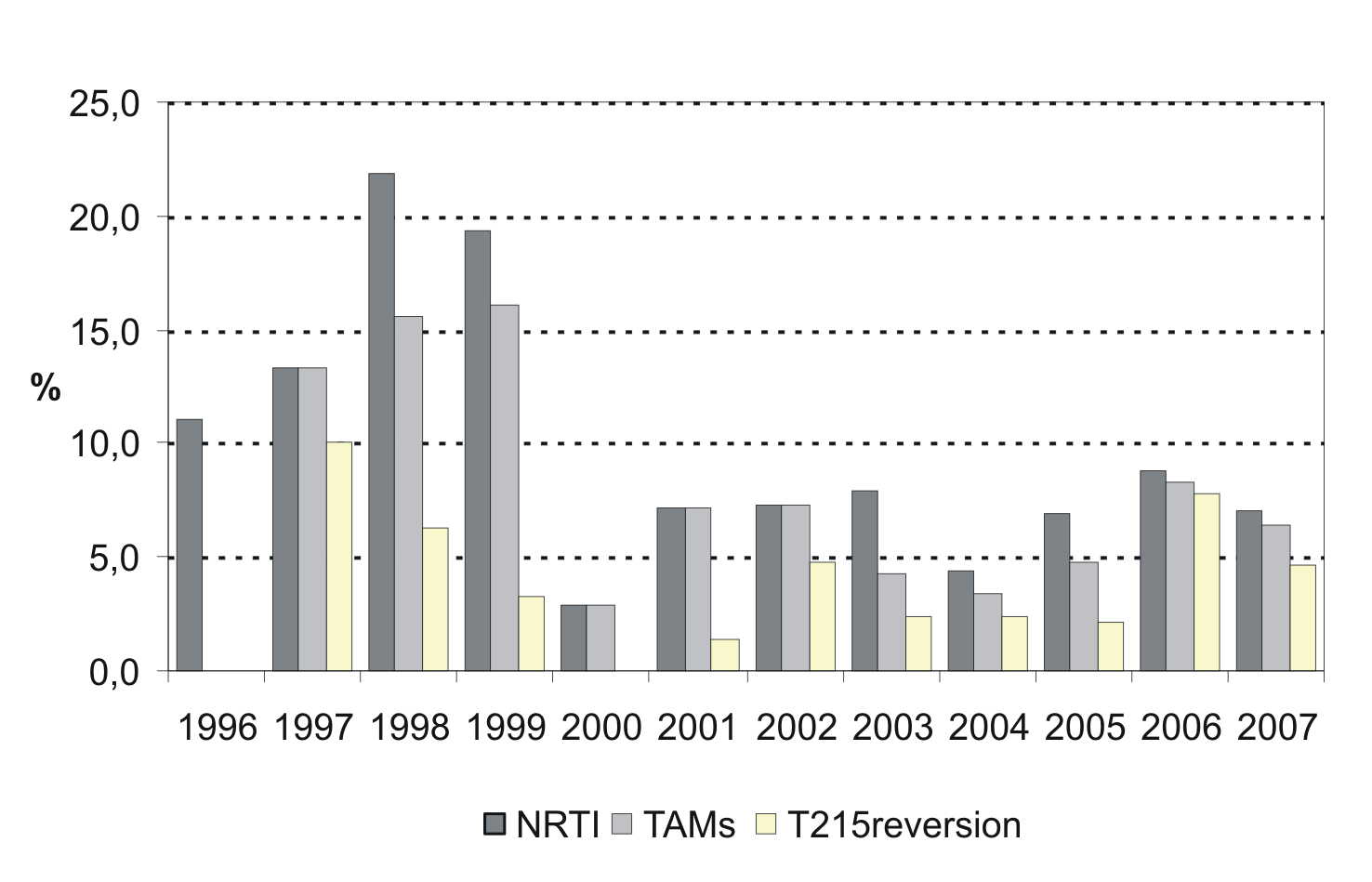

Supplement: Figure S1 — Prevalence of TAMs and NRTI resistance mutations by year of seroconversion. The prevalences of TAMs and NRTI resistance mutations (SDRM list) were calculated by year of seroconversion. (0.23 MB TIF) [file pone.0012718.s001.tif]

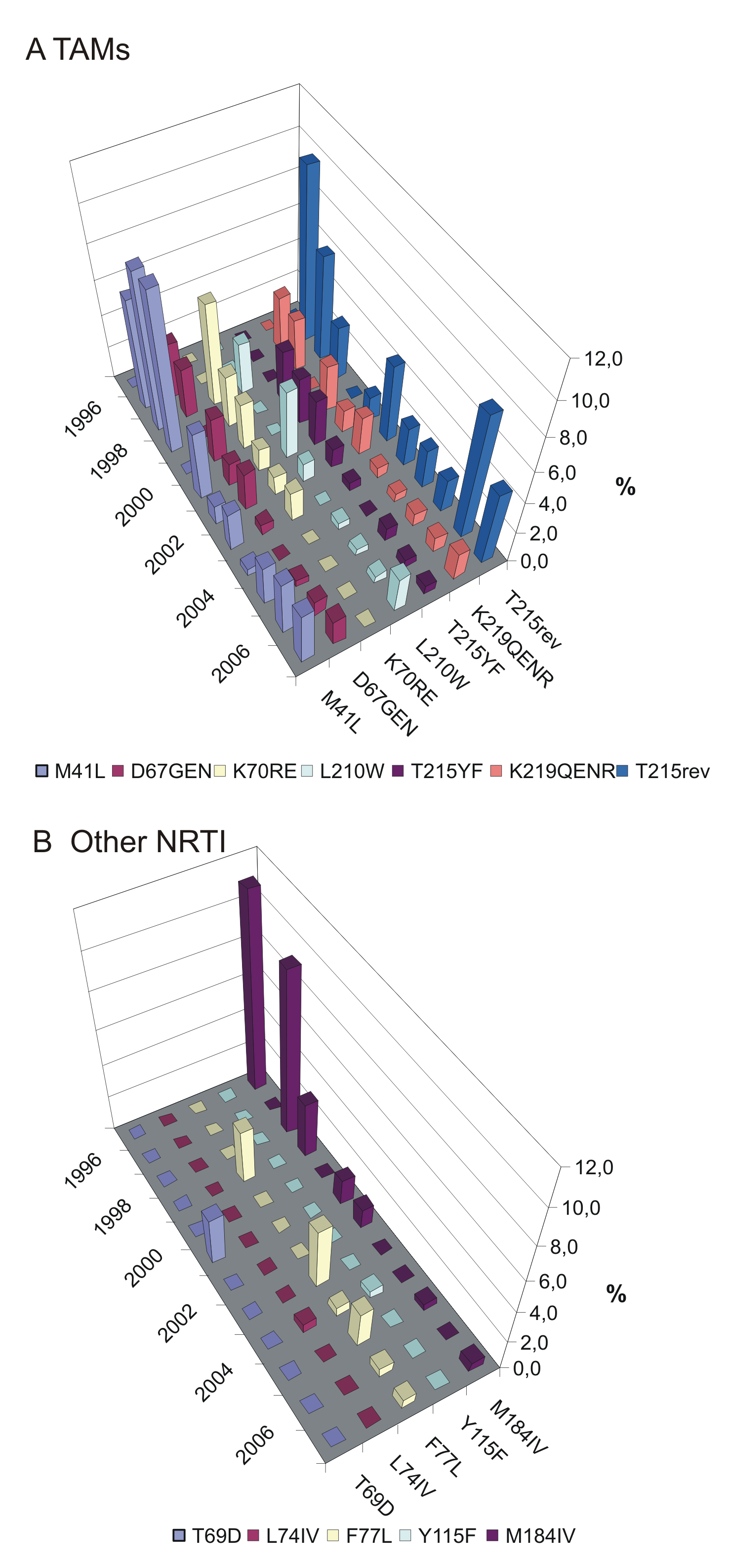

Supplement: Figure S2 — Prevalence of NRTI resistance mutations by year of seroconversion. The prevalences of each of TAMs and other NRTI resistance mutations (SDRM list) were calculated per year of seroconversion. A TAMs. Never observed: K219R. B NRTI resistance mutations other than TAMs. Never observed: K65R, T69i, L74V, V75AMTS, F116Y, Q151M. (0.88 MB TIF) [file pone.0012718.s002.tif]

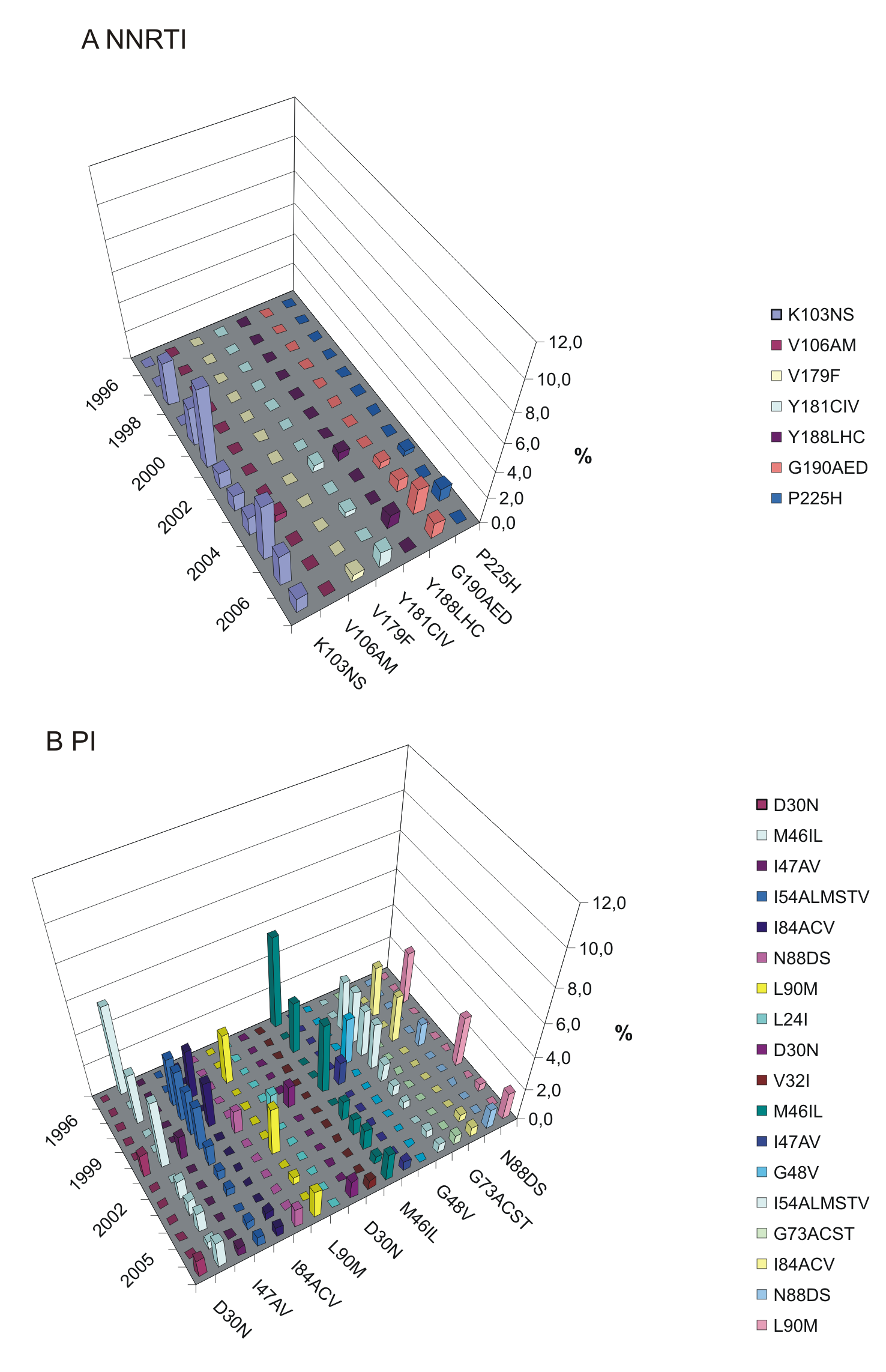

Supplement: Figure S3 — Prevalence of NNRTI and PI resistance mutations by year of seroconversion. The prevalences of each of the NNRTI and PI resistance mutations (SDRM list) were calculated by year of seroconversion. A NNRTI resistance mutations. Never observed: L100I, K101E, V106M, Y181I, G190EQ, M230L (P236L not included). B PI resistance mutations. Occurred once: L24I, V32I, G48V, G73ACST. (0.73 MB TIF) [file pone.0012718.s003.tif]

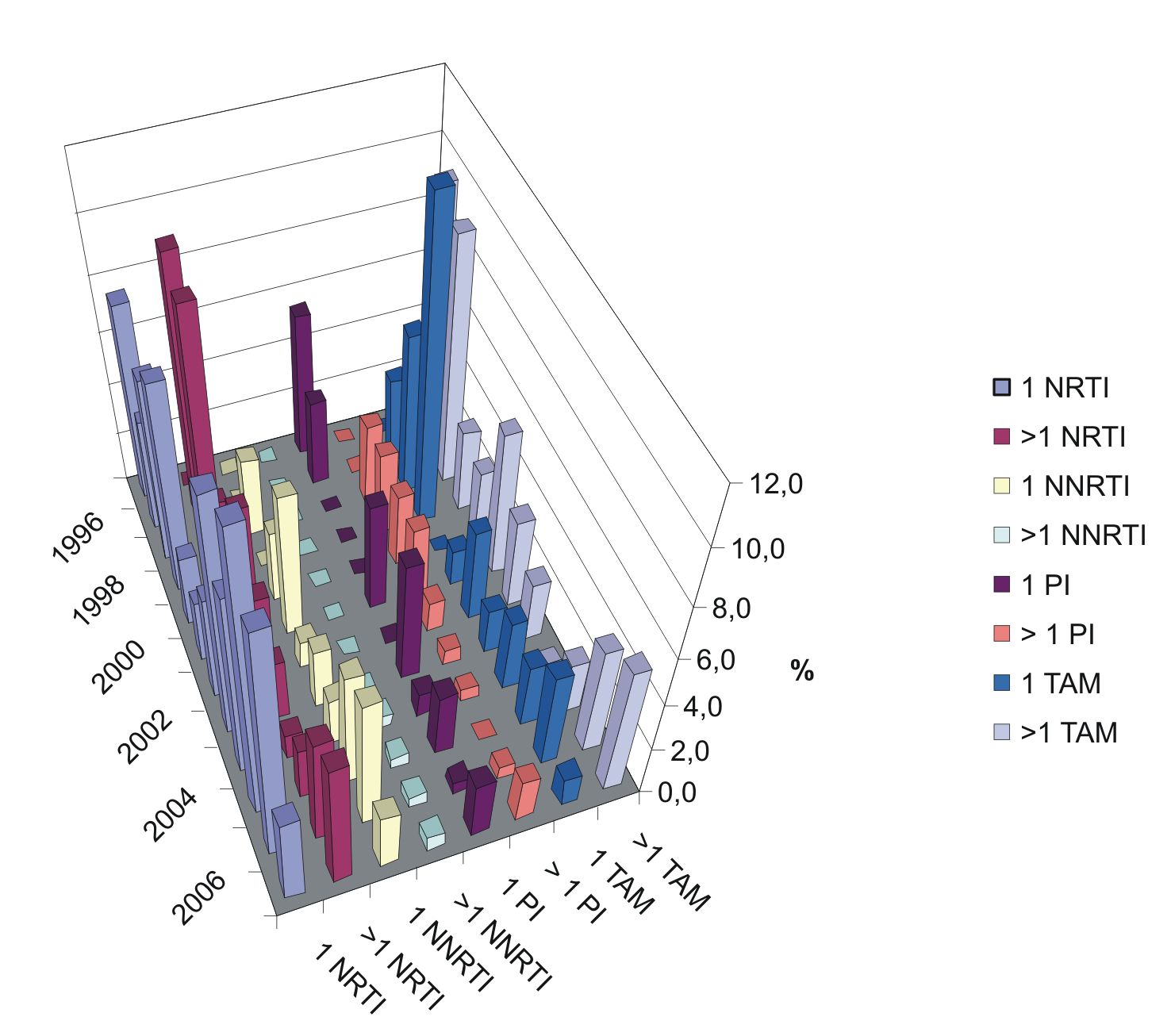

Supplement: Figure S4 — Prevalence of TDR caused by one or more resistance mutations in the HIV genome by year of seroconversion. (0.51 MB TIF) [file pone.0012718.s004.tif]
